# Methods and feasibility of collecting occupational data for a large population-based cohort study in the United States: the reasons for geographic and racial differences in stroke study

**DOI:** 10.1186/1471-2458-14-142

**Published:** 2014-02-10

**Authors:** Leslie A MacDonald, LeaVonne Pulley, Misty J Hein, Virginia J Howard

**Affiliations:** 1 National Institute for Occupational Safety and Health, 4676 Columbia Parkway, MS R15, Cincinnati, OH 45226, USA; 2 College of Public Health, University of Arkansas for Medical Sciences, 4301 West Markham Street, Little Rock, AR 72205, USA; 3 School of Public Health, University of Alabama at Birmingham, 1665 University Boulevard, Birmingham, AL 35294, USA

**Keywords:** Occupations, Occupational exposure, Stressful events, Social class, Cohort studies, Epidemiologic methods, Data collection, Stroke, Cardiovascular disease

## Abstract

**Background:**

Coronary heart disease and stroke are major contributors to preventable mortality. Evidence links work conditions to these diseases; however, occupational data are perceived to be difficult to collect for large population-based cohorts. We report methodological details and the feasibility of conducting an occupational ancillary study for a large U.S. prospective cohort being followed longitudinally for cardiovascular disease and stroke.

**Methods:**

Current and historical occupational information were collected from active participants of the REasons for Geographic And Racial Differences in Stroke (REGARDS) Study. A survey was designed to gather quality occupational data among this national cohort of black and white men and women aged 45 years and older (enrolled 2003–2007). Trained staff conducted Computer-Assisted Telephone Interviews (CATI). After a brief pilot period, interviewers received additional training in the collection of narrative industry and occupation data before administering the survey to remaining cohort members. Trained coders used a computer-assisted coding system to assign U.S. Census codes for industry and occupation. All data were double coded; discrepant codes were independently resolved.

**Results:**

Over a 2-year period, 17,648 participants provided consent and completed the occupational survey (87% response rate). A total of 20,427 jobs were assigned Census codes. Inter-rater reliability was 80% for industry and 74% for occupation. Less than 0.5% of the industry and occupation data were uncodable, compared with 12% during the pilot period. Concordance between the current and longest-held jobs was moderately high. The median time to collect employment status plus narrative and descriptive job information by CATI was 1.6 to 2.3 minutes per job. Median time to assign Census codes was 1.3 minutes per rater.

**Conclusions:**

The feasibility of conducting high-quality occupational data collection and coding for a large heterogeneous population-based sample was demonstrated. We found that training for interview staff was important in ensuring that narrative responses for industry and occupation were adequately specified for coding. Estimates of survey administration time and coding from digital records provide an objective basis for planning future studies. The social and environmental conditions of work are important understudied risk factors that can be feasibly integrated into large population-based health studies.

## Background

Although coronary heart disease (CHD) and stroke are believed to be largely preventable, they are the first and fourth leading causes of death in the United States [1]. More than half of those with cardiovascular disease (CVD) (53%) are less than 60 years old, and circulatory diseases are a leading cause of death and permanent disability among workers [2,3]. A disproportionate burden of CVD (including CHD and stroke) occurs among African Americans, low income groups, and those living in the region of the Southeastern United States known as the stroke belt [4]. Evidence increasingly suggests the need to look beyond traditional individual-level factors in understanding these patterns of risk – including the role of work [5-9]. Accordingly, several initiatives by the U.S. government seek to enhance our understanding of the social and environmental determinants of CVD through directed research efforts, including Healthy People 2020, the Public Health Action Plan to Prevent Heart Disease and Stroke, and the Occupational Health Disparities and Cardiovascular Research Programs at the National Institute for Occupational Safety and Health (NIOSH).

Evidence since the 1980s links working conditions, such as job strain and shift work, to increased risk of hypertension, heart disease [8,10-12] and stroke [13] among workers. However, much of the evidence is cross-sectional, and these studies are often small industrial cohorts that under-represent racial minorities. Population-based CVD research increasingly extends beyond traditional risk factors in an effort to understand socio-demographic differences in risk, but progress has been limited by reliance on overly-simplistic global measures of socioeconomic status (SES) [14]; occupational data collected in a majority of these studies are descriptive and used to represent SES rather than occupational exposure, affecting conclusions about where and how to direct prevention efforts [15].

We sought to advance many federal priorities in cardiovascular health research, and improve upon past studies, by designing an occupational ancillary study to supplement the extensive clinical and covariate data from an existing large cohort study. We report the methodological details and the feasibility of collecting current and historical occupational data for participants of the REasons for Geographic And Racial Differences in Stroke (REGARDS) Study.

## Methods

### Cohort

REGARDS is a longitudinal population-based cohort of 30,239 participants designed to investigate factors associated with racial and geographic differences in stroke in the United States. Inclusion in the REGARDS Study recruitment sample required having a name, telephone number and address in a commercially available nationwide list of households in the United States, which is routinely updated from multiple sources (e.g., telephone directories, motor vehicle registrations, real estate listings, and driver’s license data) [16]. Recruitment involved a stratified random sample balanced by race, sex, and geographic regions of the stroke belt (defined as the eight southern states of North Carolina, South Carolina, Georgia, Tennessee, Mississippi, Alabama, Louisiana, and Arkansas) and the remaining 40 contiguous states. English-speaking community-dwelling individuals aged 45 years or older who self-identified as non-Hispanic black or white were invited to participate, and those who were eligible and consented completed a telephone interview and an in-home medical examination January 2003-October 2007. Exclusion criteria included self-reported medical conditions (such as cancer) that would prevent long-term participation, or being on a waiting list for a nursing home. Complete details of the study recruitment methodology, selection criteria, and participation rates are available elsewhere [17,18].

At the conclusion of participant enrollment, the demographic distribution of the cohort was 42% black and 55% women; the geographical distribution was 56% from the stroke belt, and 44% from the remaining states [18]. Participants are being followed by telephone at six-month intervals for incident stroke events and change in cognitive functioning. Ancillary studies have broadened goals to assess racial and geographic differences in other cardiovascular outcomes including heart disease [19], kidney disease [20], and venous thrombosis [21].

### Funding, collaboration, and scope

The National Institutes of Health (NIH), National Institute for Neurological Disorders and Stroke (NINDS), provided initial funding for establishment of the REGARDS Study cohort and telephone follow-up (2001–2007 and 2007–2012). Recently, NINDS awarded additional funding to conduct a second in-home physical exam, an extensive CVD telephone survey and continuation of telephone follow-ups (2012-2017). Employment status was collected at enrollment; however, no data were obtained on workplace conditions that may increase risk of CVD (e.g., shift work). An ancillary study proposal for an occupational supplement was approved by the REGARDS Executive Committee, and developed into an intramural research application at NIOSH. NIOSH funding was awarded in 2010 for development and administration of an occupational survey to the REGARDS cohort. The study was approved by the Institutional Review Boards at NIOSH and the University of Alabama at Birmingham (UAB).

### Occupational survey

All active members of the REGARDS cohort were targeted for administration of the occupational survey by Computer-Assisted Telephone Interview (CATI) during routine bi-annual follow-up. The survey was organized into six sections: employment status, entire working career, current job, current job exposures, longest-held job, and the job held at REGARDS enrollment (“enrollment job”). The survey content areas are summarized in Table 1 for each section of the survey. The CATI system automated the order of questions in accordance with prescribed skip patterns. Participants completing the occupational survey reported their current employment status (March 2011-March 2013) and retrospectively reported employment status at REGARDS Study enrollment (2003–2007). Only participants employed outside the home at 25 years of age or older were asked to report occupational information. Study resources dictated that we aim to keep administration time to 10 minutes on average, so the survey was designed to avoid repeat reporting of job details. Participants first reported information about their current job and associated workplace exposures; they were then asked about their longest-held job only if it was different from the current job; finally, they were asked questions about the job held at enrollment only if this job was different from the current and/or longest-held jobs. Thus, each participant reported characteristics for up to three jobs – current, longest-held, and enrollment.

**Table 1:** REGARDS occupational survey content areas

	
**Employment Status**	**Longest-Held Job (self-employed)**
• At enrollment	• Same as enrollment job
• Current	• Same as current job
**Entire Work Career**	• Industry
• Ever worked shiftwork	• Job title and duties
• Years of shiftwork	• Job tenure
• Type of shiftwork	• Year left this job
• Number of jobs held in the past 10 years	• Employer size
**Current Job (self-employed)**	• Work arrangement
• Industry	• Union status
• Job title and duties	• Supervisory duties
• Start year	• Total work hours per week
• Employer size	• (Number of employees)
• Work arrangement^a^	• (Income ≥ 20% household income)
• Union status	**Enrollment Job (self-employed)**
• Supervisory duties	• Same as longest-held job
• (Number of employees)	• Same as current job
• (Income ≥ 20% household income)	• Industry
**Current Job Exposures**	• Job title and duties
• Shiftwork	• Job tenure
• Total work hours per week	• Year left this job
• Work hour preference	• Employer size
• Psychological job demands^b^	• Work arrangement
• Decision latitude^b^	• Union status
• Skill discretion^b^	• Supervisory duties
• Overall physical effort level^c^	• Total work hours per week
• Work-life imbalance^d^	• (Number of employees)
• Threatened or bullied in the past year^e^	• (Income ≥ 20% household income)
• Discrimination (age, race, gender, other)^e^	

^a^Work arrangement categories for wage employed participants included: on-call employee, subcontractor or employee of a temporary agency, regular permanent employee, and unknown; work arrangement categories for self-employed participants included: business owner, independent contractor or consultant or freelance worker.

^b^Job Content Questionnaire scale measures [22].

^c^One item modified Borg perceived exertion scale [23].

^d^One item adapted from a validated scale based on expert consultation with Dr. Joseph Grzywacz [24].

^e^NIOSH Quality of Work Life Survey [25].

### Pilot study

The occupational survey was pilot tested in October 2010 by the Survey Research Unit (SRU) at UAB to evaluate CATI administration procedures, administration time, and data quality. Following a standard interviewer orientation to the new survey, 106 randomly selected REGARDS Study participants were contacted, ninety-one of whom consented (91/106 = 86% response rate). Mean and median administration times were approximately 8 minutes (range 1–23 minutes). Some technical problems involving skip patterns in the CATI system were identified and resolved shortly after the pilot began; other skip pattern problems were identified after pilot data collection was completed, resulting in extensive re-programming and testing prior to full-scale administration. A NIOSH expert in Industry and Occupation (I/O) coding reviewed all narrative responses for industry and occupation (i.e., industry type and job title/kind of work) from the pilot surveys (81 jobs: 19 current and 62 longest-held), and determined that data quality was inadequate for assigning 4-digit Census codes for 12% of the jobs. Data quality problems included typographical errors, abbreviation usage, insufficient detail, and the reporting of multiple job titles.

To improve the quality of I/O data collection and subsequent coding, a 2-day in-person interviewer training was conducted before administration of the survey to the remaining cohort. The training provided numerous examples of “adequate” and “inadequate” text descriptions for industry, job title and job duties, as described in two NIOSH documents [26,27]. The interviewers were instructed when and how to probe study participants to elicit more specific responses when their initial reply was inadequate for coding purposes. For example, if a participant reported a job title of “teacher” or “manager,” interviewers were instructed to probe for the more specific response of “high school teacher” or “grocery store manager”. The training also emphasized the importance of correct spelling, not using abbreviations, and reporting only one job (the job requiring the most work hours for participants who held multiple jobs concurrently).

### Data collection

Highly trained interviewers at the SRU routinely conduct approximately 3,500 REGARDS Study follow-up interviews each month. The occupational survey was included as a module within these routine follow-up interviews. Full-scale administration to the remainder of the active cohort was conducted March 2011 through March 2013. Study participants were notified about the new survey in advance through the REGARDS Study newsletter. During the follow-up call, verbal consent to complete the occupational survey was obtained prior to administration. When a participant was unavailable during repeated call attempts, the survey was administered during the subsequent follow-up period.

### Industry and occupation coding

Throughout data collection, de-identified data from the occupational survey were periodically transferred to NIOSH through a secured File Transfer Protocol. Once received, a file of the narrative industry and occupation data was prepared for import into the NIOSH Industry and Occupation Computer-Assisted Coding System [28]. This coding system was developed by NIOSH to aid trained coders in the efficient assignment of standardized industry and occupation codes. A double-coding procedure was used in which each imported file was assigned to two raters from a pool of three. Each rater independently reviewed narrative information for each job and assigned 4-digit U.S. Census 2002 codes for industry and occupation; a fourth rater resolved discrepancies. Additional quality assurance was performed on the first 52% of the records by a NIOSH I/O coding expert who reviewed participants’ narrative data any time a unique combination of industry and occupation looked unusual.

### Statistical methods

All statistical analyses were performed using SAS (version 9.3, SAS Institute Inc., Cary, NC). Reliability of the initial assigned I/O codes was assessed using percent agreement; inter-rater agreement was evaluated for an exact match on the assigned 4- (and 2-) digit Census codes for both industry and occupation. The un-weighted kappa statistic was used to assess concordance of the current and longest-held jobs with respect to industry and occupation. These results were stratified by age at the occupational survey (<65 years, 65+ years). Time estimates for survey completion, and for sub-sets of survey items, were computed from digital time-stamp data extracted from the CATI system. Time estimates for assigning industry and occupation codes were computed from digital time-stamp data extracted from the NIOSH computer-assisted coding system.

## Results

### Survey administration

**Figure 1:**
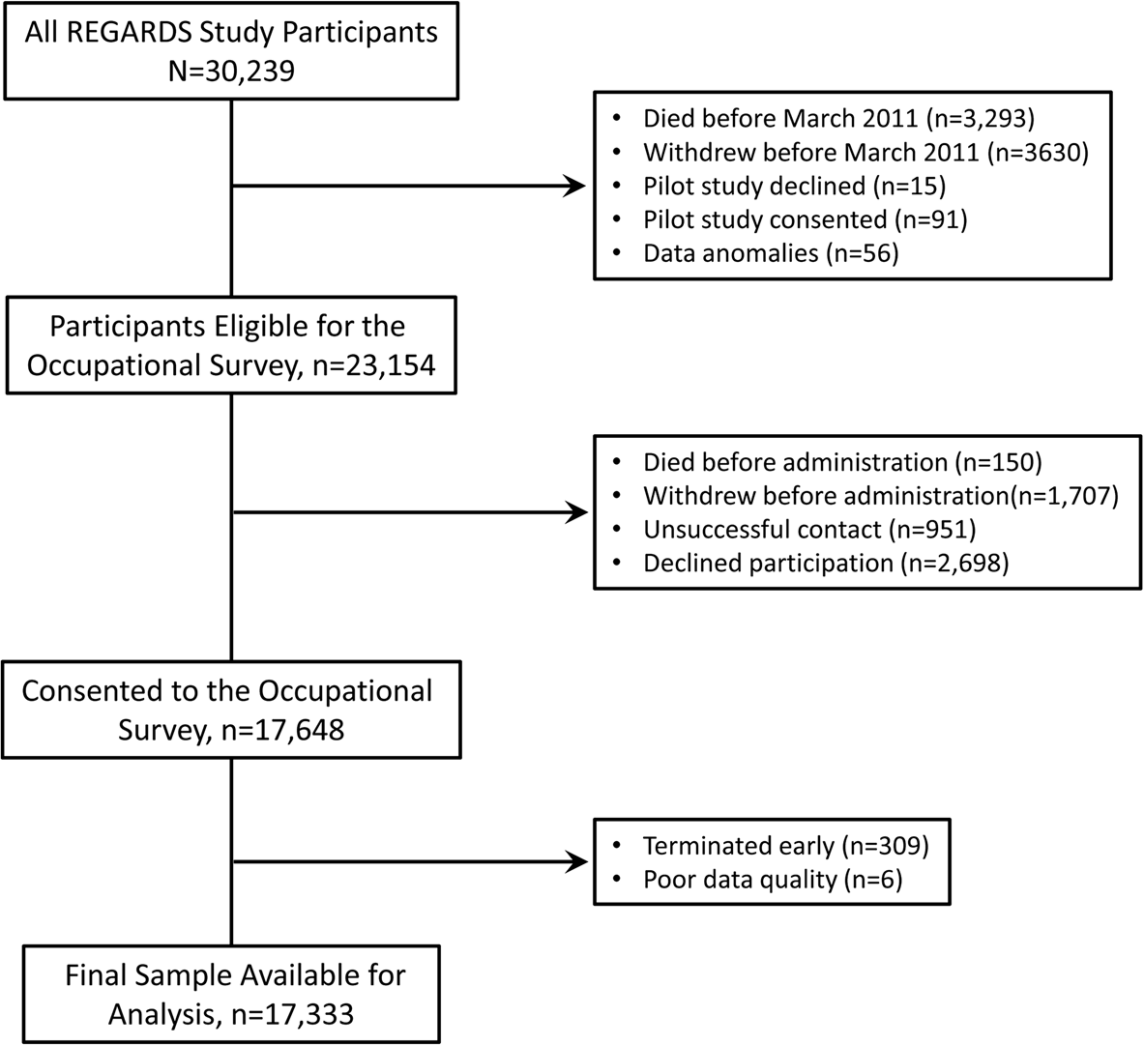
REGARDS occupational study sample size tracing.

**Figure 2:**
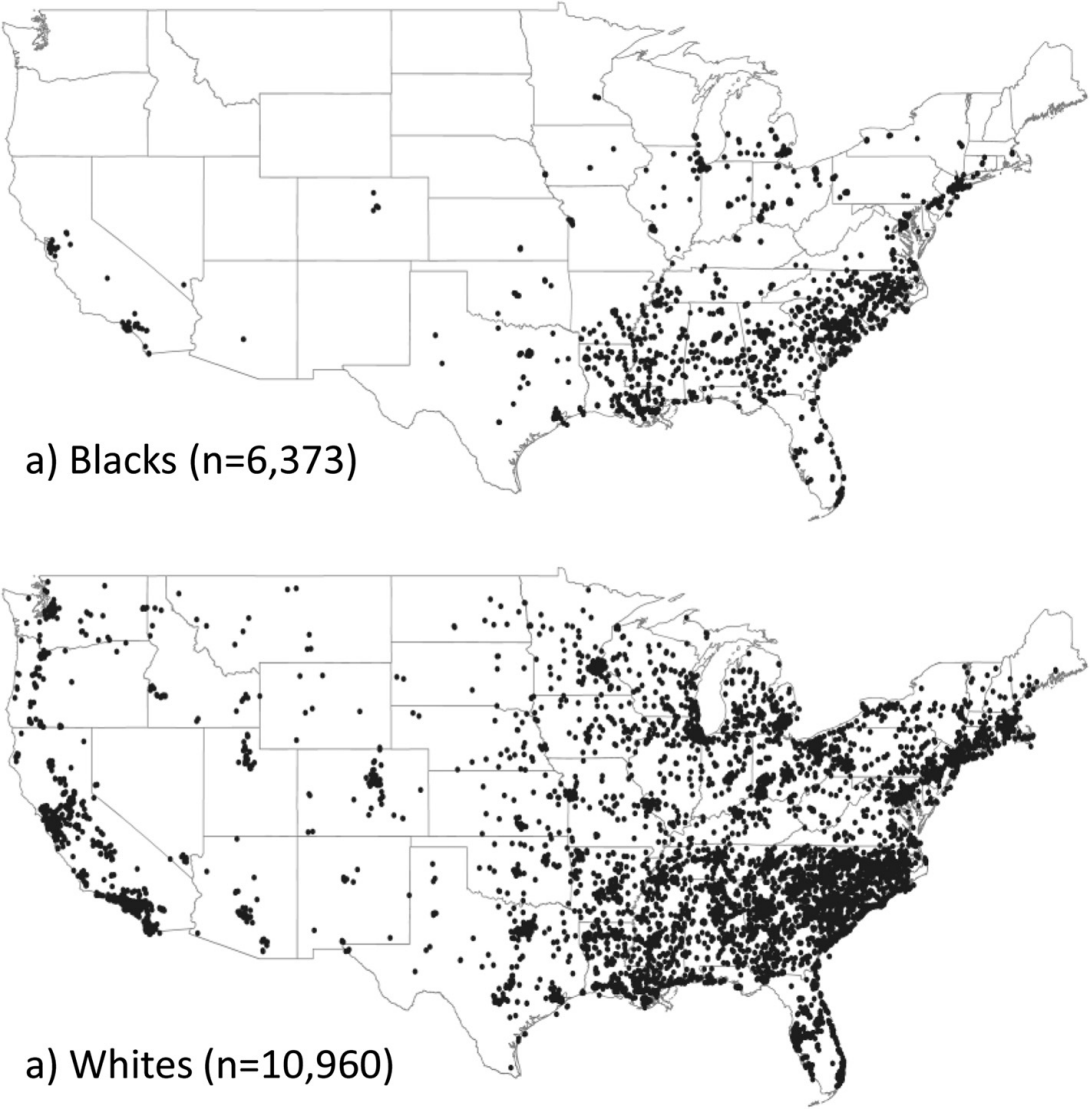
Geographic distribution of REGARDS occupational study sample for a) blacks and b) whites, N = 17,333.

The occupational survey was administered 3.5 to 9.5 years after participant enrollment. Of the 30,239 original REGARDS participants, 77% (n = 23,154) were eligible to complete the survey in March 2011, after accounting for those selected for the pilot (n = 106), those who had withdrawn from the study or died (n = 6,923), and those with data anomalies (n = 56). Among the eligible participants, 88% were contacted to complete the survey during the two-year administration period. Among those not contacted, 1,857 participants died or withdrew before the survey could be administered and 951 were not reached after multiple call attempts. Among participants who were contacted, 17,648 consented (17,648/20,346 = 87% response rate). Participants with poor data quality (n = 6) or who consented to the occupational survey but terminated early or otherwise refused to answer questions (n = 309) were excluded from further analysis. The final number of completed surveys available for analysis was 17,333 (Figure 1). The geographic distributions of the final sample by race are shown in Figure 2.

**Figure 3:**
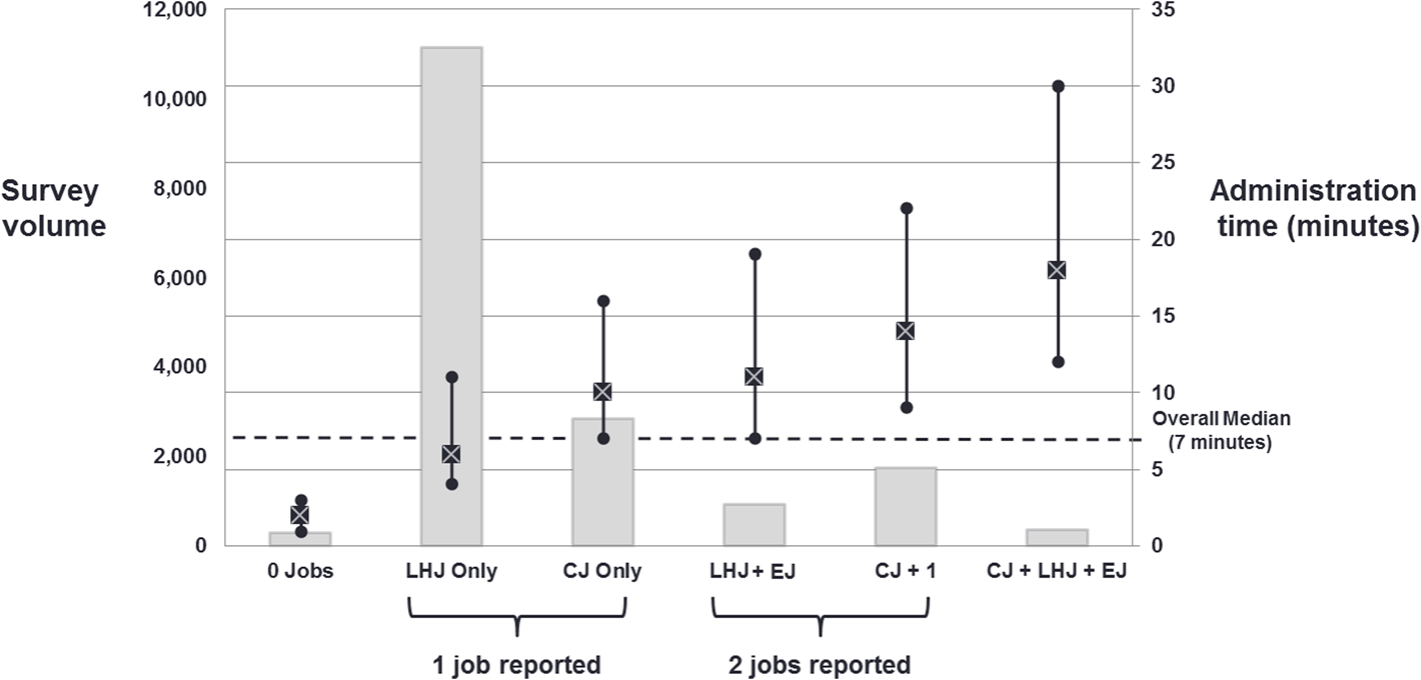
Survey volume and total administration time (median and 5th and 95th percentiles) by the number of jobs reported (LHJ: longest-held job, CJ: current job, EJ: Enrollment job).

Overall median administration time for completing the entire occupational survey was 7 minutes, and varied according to the number and type of jobs reported (Figure 3). Median administration time was lowest (2 minutes) for a small number of participants who reported no job-specific details. For the majority of participants (n = 11,148) who only reported a longest-held job, median administration time was 6 minutes. Median administration time was 4 minutes longer (10 minutes total) for participants only reporting a current job (and current exposures). Median participation time was more than the 10 minute target for the minority of participants who reported 2 or 3 jobs. The median administration time for collecting only narrative information required for industry and occupation coding (i.e., employment status, job title (wage-employed)/kind of work (self-employed), industry type) ranged from 0.72 minutes (43 seconds) for participants reporting a current wage job to 1.5 minutes for those reporting a longest-held job in which they were self-employed. This median time increased less than one minute to a range of 1.6 to 2.3 minutes, respectively, when the following additional job information was collected: “main job activities or duties” and “supervisory responsibilities (wage-employed)”/“number of employees (self-employed)”.

### Sample characteristics

Demographic and descriptive characteristics for the occupational study sample are shown in Table 2, by race and sex. Most participants (68%) had more than a high school education at enrollment; at the time of the occupational survey, most were age 65 years or older (71%) and retired (61%). Nearly one quarter (23%) of the retirees had left employment within the past 5 years. Twenty-nine percent reported a current job at the time of the occupational survey, down from 43% who were employed at enrollment. A small number of participants (n = 277, 2%) reported not working outside the home after the age of 25. A total of 7% reported being currently unemployed or unable to work.

**Table 2:** **Characteristics of the REGARDS occupational ancillary study sample (n = 17,333)**^**a**^

**Characteristic**	**Black women (n = 4,086)**	**Black men (n = 2,287)**	**White women (n = 5,563)**	**White men (n = 5,397)**	**Overall (n = 17,333)**
**Age at the occupational survey (y)**					
45-54	214 (5)	124 (5)	322 (6)	196 (4)	856 (5)
55-64	1167 (29)	522 (23)	1456 (26)	1047 (19)	4192 (24)
65-74	1674 (41)	966 (42)	2168 (39)	2289 (42)	7097 (41)
75 or older	1031 (25)	675 (30)	1617 (29)	1865 (35)	5188 (30)
**Education at enrollment**					
Unknown	3 (<1)	0 (0)	2 (<1)	1 (<1)	6 (<1)
High school or less	1663 (41)	917 (40)	1700 (31)	1295 (24)	5575 (32)
Some college	1164 (28)	625 (27)	1595 (29)	1274 (24)	4658 (27)
College graduate and above	1256 (31)	745 (33)	2266 (41)	2827 (52)	7094 (41)
**US region at enrollment**					
Stroke buckle^b^	816 (20)	408 (18)	1485 (27)	1085 (20)	3794 (22)
Rest of stroke belt	1399 (34)	787 (34)	1930 (35)	1809 (34)	5925 (34)
Other 40 contiguous states	1871 (46)	1092 (48)	2148 (39)	2503 (46)	7614 (44)
**Residence at enrollment**^**c**^					
Unknown	326 (8)	152 (7)	620 (11)	545 (10)	1643 (9)
Rural	504 (12)	244 (11)	1280 (23)	1150 (21)	3178 (18)
Urban	3256 (80)	1891 (83)	3663 (66)	3702 (69)	12512 (72)
**Employment status at the occupational survey**					
Currently employed	1057 (26)	622 (27)	1601 (29)	1669 (31)	4949 (29)
Retired^d^	2425 (59)	1457 (64)	3157 (57)	3502 (65)	10541 (61)
Unemployed/unable to work	474 (12)	201 (9)	310 (6)	212 (4)	1197 (7)
Homemaker or student	128 (3)	5 (<1)	489 (9)	11 (<1)	633 (4)
Not working, reason unknown	1 (<1)	2 (<1)	6 (<1)	2 (<1)	11 (<1)
Unknown	1 (<1)	0 (0)	0 (0)	1 (<1)	2 (<1)
**Ever employed outside the home ≥ 25 years old**					
Unknown	6 (<1)	1 (<1)	8 (<1)	0 (0)	15 (<1)
No	72 (2)	14 (<1)	178 (3)	13 (<1)	277 (2)
Yes	4008 (98)	2272 (99)	5377 (97)	5384 (100)	17041 (98)
**Employed at REGARDS enrollment?**					
Unknown	4 (<1)	8 (<1)	9 (<1)	11 (<1)	32 (<1)
No	2437 (60)	1262 (55)	3264 (59)	2806 (52)	9769 (56)
Yes	1645 (40)	1017 (44)	2290 (41)	2580 (48)	7532 (43)

^a^All values are n (percentage). Percentages may not sum to 100 due to rounding.

^b^The stroke buckle is a segment of the stroke belt region of the United States defined as the south Atlantic coastal plains states of North Carolina, South Carolina and Georgia.

^c^Classification based on Rural–urban Community Area (RUCA) codes.

^d^Median years since retirement 11.0 years (range 0–60 years).

### Industry and occupation coding

**Figure 4:**
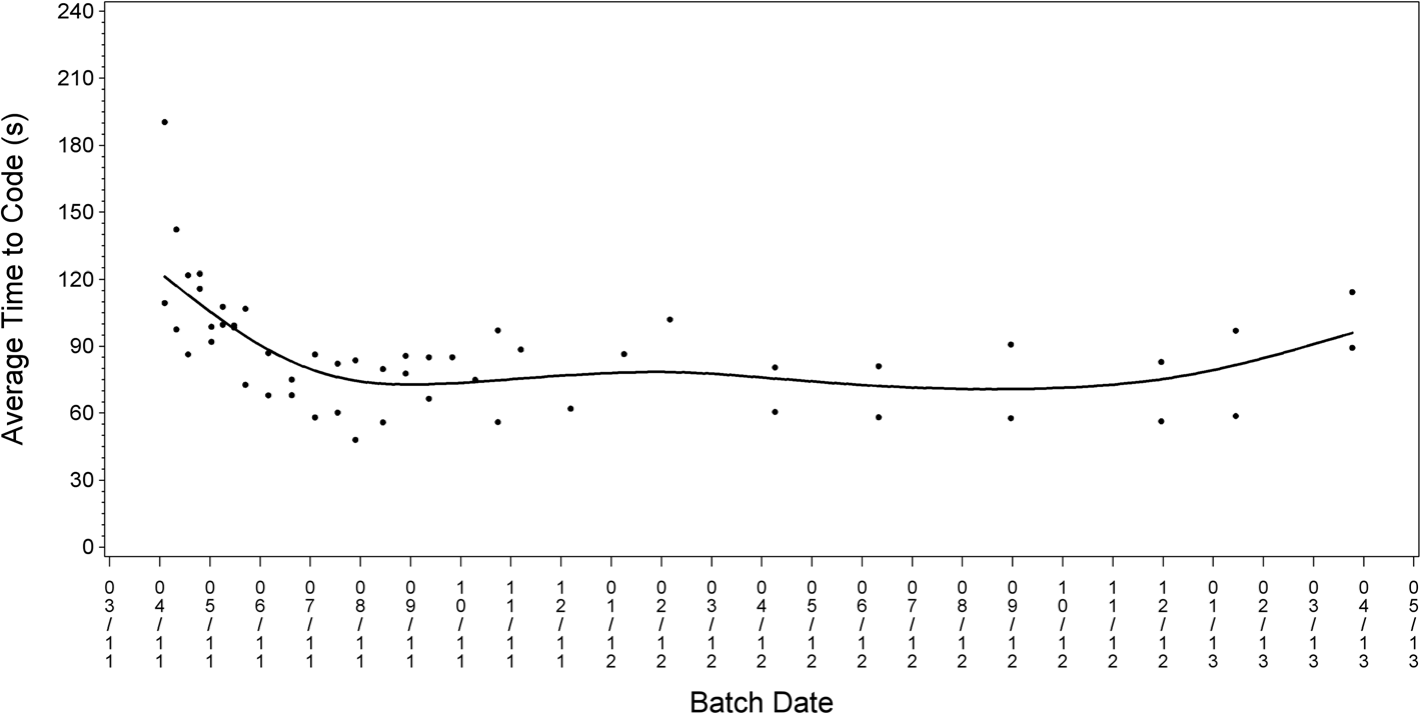
The average time to code industry and occupation in seconds (s) by batch date. Each symbol (●) indicates a batch-coder. The smooth curve was fit using a spline routine.

A total of 20,427 jobs were assigned 4-digit Census codes for industry and occupation. Inter-rater agreement at the 4-digit level was 80% for industry, 74% for occupation, and 61% for both codes; agreement at the 2-digit level was 83%, 78%, and 73%, respectively. There was no evidence of a training effect between the initial and subsequent batches of coded data. A review of the unique I/O combinations encountered in the first half of data collection indicated very few unusual combinations (27 out of 3,609 I/O combinations from 10,791 jobs) and even fewer jobs with possible coding errors (9 or <0.1%). Overall, less than 0.5% of the narrative data were uncodable (Table 3). Average time to code both industry and occupation was 1.3 minutes per job per rater; higher coding time for earlier batches likely reflects a learning curve (Figure 4).

**Table 3:** **Characteristics of jobs reported by the REGARDS occupational ancillary study sample, by job type**^**a**^

**Characteristic**	**Job type**^**b**^		
	**Current (n = 4,949)**	**Longest held (n = 17,041)**	**Enrollment (n = 7,532)**
**Type of employment**			
Unknown	0 (0)	18 (<1)^c^	823 (11)^d^
Wage	3638 (74)	14988 (88)	5254 (70)
Self	1311 (26)	2035 (12)	1455 (19)
**Among wage employed**			
Covered by a union?	507 (14)	3824 (26)	1043 (20)
Supervisory responsibilities?	1283 (35)	7777 (52)	2351 (45)
**Work arrangement**			
On call employee	172 (5)	166 (1)	124 (2)
Subcontractor/temporary agency	109 (3)	135 (1)	91 (2)
Regular, permanent employee	3326 (91)	14626 (98)	5012 (95)
Unknown	31 (1)	61 (<1)	27 (1)
**Among self employed**			
Self-employment wages represent ≥20% of household income?	852 (65)	1553 (76)	1068 (73)
** Work arrangement**			
Business owner	988 (75)	1630 (80)	1150 (79)
Independent contractor/consultant/freelance	323 (25)	405 (20)	305 (21)
**Industry sector (census based)**^**e**^			
Unknown	0 (0)	18 (<1)	823 (11)
Not codable	2 (<1)	9 (<1)	12 (<1)
Agriculture, forestry & fishing	75 (2)	196 (1)	92 (1)
Construction	197 (4)	577 (3)	277 (4)
Health care & social assistance	817 (17)	2280 (13)	1014 (13)
Manufacturing	338 (7)	2867 (17)	656 (9)
Mining	10 (<1)	62 (<1)	17 (<1)
Services^f^	2799 (57)	8405 (49)	3666 (49)
*Information*	*97 (2)*	*567 (3)*	*142 (2)*
*Finance and insurance*	*216 (4)*	*722 (4)*	*293 (4)*
*Real estate, rental, and leasing*	*188 (4)*	*266 (2)*	*209 (3)*
*Professional, scientific, and technical*	*372 (8)*	*654 (4)*	*455 (6)*
*Corporate management*	*0 (0)*	*6 (<1)*	*1 (<1)*
*Administrative support and waste management*	*162 (3)*	*284 (2)*	*209 (3)*
*Education*	*791 (16)*	*2884 (17)*	*1129 (15)*
*Arts, entertainment, and recreation*	*122 (2)*	*150 (1)*	*111 (1)*
*Accommodation and food*	*69 (1)*	*315 (2)*	*98 (1)*
*Public administration (government)*	*348 (7)*	*1679 (10)*	*509 (7)*
*Other service industries*	*434 (9)*	*878 (5)*	*510 (7)*
Transportation, warehousing & utilities	239 (5)	1105 (6)	390 (5)
Wholesale and retail trade	470 (9)	1275 (7)	582 (8)
Armed forces	2 (<1)	247 (1)	3 (<1)
**Occupational group**^**g**^			
Unknown	0 (0)	18 (<1)	823 (11)
Not codable	4 (<1)	10 (<1)	12 (<1)
11-Management	633 (13)	2359 (14)	908 (12)
13-Business and financial operations	277 (6)	684 (4)	352 (5)
15-Computer and mathematical science	86 (2)	250 (1)	126 (2)
17-Architecture and engineering	97 (2)	467 (3)	151 (2)
19-Life, physical, and social science	72 (1)	256 (2)	101 (1)
21-Community and social services	206 (4)	518 (3)	261 (3)
23-Legal	89 (2)	174 (1)	121 (2)
25-Education, training, and library	544 (11)	2039 (12)	771 (10)
27-Arts, design, entertainment, sports, and media	167 (3)	274 (2)	175 (2)
29-Healthcare practitioner and technical	338 (7)	1067 (6)	439 (6)
31-Healthcare support	115 (2)	363 (2)	130 (2)
33-Protective service (firefighters/law enforcement)	121 (2)	318 (2)	151 (2)
35-Food preparation and serving related	78 (2)	343 (2)	104 (1)
37-Building and grounds cleaning and maintenance	139 (3)	386 (2)	208 (3)
39-Personal care and service	227 (5)	386 (2)	229 (3)
41-Sales and related	504 (10)	1192 (7)	647 (9)
43-Office and administrative support	618 (12)	2520 (15)	850 (11)
45-Farming, fishing, and forestry	17 (<1)	54 (<1)	23 (<1)
47-Construction and extraction	123 (2)	464 (3)	190 (3)
49-Installation, maintenance, and repair	104 (2)	540 (3)	139 (2)
51-Production	171 (3)	1438 (8)	314 (4)
53-Transportation and material moving	219 (4)	686 (4)	304 (4)
55-Military	0 (0)	235 (1)	3 (<1)

^a^All values are n (percentage). Percentages may not sum to 100 due to rounding.

^b^Current jobs were held when the NIOSH survey was administered; longest-held jobs include 2883 current jobs that were the same as the longest-held job; and enrollment jobs were held at REGARDS enrollment and include 3454 current and 2276 longest-held jobs that were the same as the enrollment job (see Additional file 1).

^c^Unknown for 18 subjects that reported working for pay at the REGARDS enrollment interview, but did not report any employment at the NIOSH occupational survey.

^d^Unknown for 823 subjects that reported working for pay at the REGARDS enrollment interview (based on concurrent reports for 539 subjects and retrospective reports for 284 subjects), but did not report any job at the NIOSH occupational survey that coincided with the enrollment interview date (see Additional file 1).

^e^US 2002 Census codes for industry were aggregated to 8 industry sectors.

^f^Subcategories of the service industry are reported in italics.

^g^US 2002 Census codes for occupation were aggregated to 23 standard major occupational groups. The two-digit notation refers to the first two digits of the Standard Occupation Classification.

### Occupation and industry profile of the sample

After participant job reporting was reviewed for ascertainment of the longest-held and enrollment jobs (see Additional file 1), a total of 29,522 jobs were identified: 4,949 current, 17,041 longest-held, and 7,532 at enrollment. Descriptive characteristics of all three jobs are shown in Table 3. Across all reported jobs, most participants were (or had been) employed for wages in a regular permanent job. Approximately one-quarter of currently employed participants were self-employed (26%); most currently self-employed participants reported contributing more than 20% of household income (65%) and being business owners (75%). Participants were represented in every Census 2002 industry code (270 codes) and nearly every Census 2002 occupation code (479 out of 506 codes).

Concordance between the current and longest-held jobs was moderately high across occupational categories (93, 22, and 6 categories), with kappa values ranging from 0.64 to 0.68, respectively for the overall sample (Table 4). Concordance was slightly higher among those less than 65 years old, compared with those 65 years and older. A similar pattern of results was found for industry.

**Table 4:** Concordance of the current job and longest held job, overall and by age

**Classification**	**Overall (n = 4,904)**^**a**^	**Age < 65 years (n = 2,777)**	**Age ≥ 65 years (n = 2,127)**
	**κ (95% CI)**	**κ (95% CI)**	**κ (95% CI)**
**Industry**			
Simple (20 groups)	0.67 (0.65-0.68)	0.71 (0.70-0.73)	0.60 (0.58-0.62)
Detailed (78 groups)	0.64 (0.63-0.66)	0.69 (0.67-0.71)	0.58 (0.56-0.61)
**Occupation**			
Broad (6 groups)	0.68 (0.67-0.70)	0.73 (0.71-0.75)	0.63 (0.60-0.65)
Simple (22 groups)	0.67 (0.66-0.68)	0.72 (0.70-0.73)	0.61 (0.58-0.63)
Detailed (93 groups)	0.64 (0.63-0.66)	0.70 (0.68-0.71)	0.58 (0.55-0.60)

*Abbreviations:κ* kappa coefficient, *CI* confidence interval.

^a^Limited to participants with known industry and occupation codes for both the current and longest-held jobs, but excludes participants with jobs in the military.

## Discussion

We report the methodological details and feasibility of collecting current and historical occupational data among an established middle-aged and older cohort 3.5-9.5 years after their enrollment in the REGARDS Study. The industry and occupational profile of the REGARDS cohort was unknown but presumed to be heterogeneous at the outset; data collection efforts therefore focused on gathering descriptive work histories (i.e., industry and occupation for the current, longest-held and enrollment jobs) that were subsequently assigned Census codes, and general job characteristics (e.g., employer size, work arrangement or contract, work schedule demands). Measures of specific workplace psychosocial stressors (e.g., job strain, discrimination) were also obtained for those still employed. Although only 29% of this large cohort was currently employed, data on the longest-held and enrollment jobs will enable future analyses involving individuals who are now retired and unemployed.

The pilot phase of occupational data collection was critical for highlighting the need for additional CATI system programming and interviewer training. Subsequent findings demonstrated that interviewer training substantially improved the quality of I/O data collection used to assign Census codes: the proportion of uncodable jobs was reduced from 12% during the pilot to less than 0.5% for the remainder of the cohort.

### Strengths

Assigning standardized I/O codes from narrative responses is a labor-intensive activity, and participants in this study reported more than 20,000 jobs that were subsequently assigned Census codes. To efficiently manage such a large volume of coding we employed the use of a computer-assisted coding system developed at NIOSH. This coding system eliminated the need for coders to manually sort through large alphabetized coding manuals in search of codes for each narrative industry and occupation description. Instead, the system displayed narrative responses from participants, along with text-matched descriptions (and corresponding codes) that coders could select from or perform additional coder-specified queries. The coding efficiency achieved through the use of this system enabled us to improve coding reliability by employing a protocol in which all jobs were coded twice by independent coders. When assigned codes did not match at the 4-digit level, discrepancies were resolved by a more senior coder. Coder reliability in our study was slightly better than previous reports [29]. We believe this reliability improvement was due to our collecting and providing supplemental job data to the coders; these data included “job duties or activities” and “supervisory responsibilities”. Favorable results from a quality assurance step provided additional confidence in the quality of the final assigned codes.

I/O codes serve several important functions in the context of population-based health research. I/O codes and their corresponding aggregate groupings (e.g., management occupations) can serve as a descriptive index of socioeconomic status. However, even greater public health value resides in the use of I/O codes to describe health patterns and to examine relationships between associated (modifiable) job characteristics and health outcomes. Describing patterns of chronic health by industry and occupation can help identify groups in need of targeted health promotion and health protections. Additionally, I/O codes can be used to link health records to archival exposure data for conducting epidemiologic analyses. Although health-exposure linkage using industry and occupation codes is a well-established methodology [30,31], a recent survey found this practice to be greatly underutilized in population-based CVD studies in the United States [15].

### Limitations

Practical considerations related to budget and participant burden made it necessary to design the survey to take no more than 10 minutes on average to administer by CATI. We therefore had to balance the need for work history coverage and depth. The collection of data on exposures was restricted to the current job because historical reports of occupational exposure to job strain have been found to be too unreliable [32]. Furthermore, a participant who held multiple jobs concurrently only reported the job where they spent the majority of their working hours, so their exposure information may be incomplete. While we were constrained in our ability to obtain a complete work history, we were able to obtain data on the longest-held and/or the enrollment job because of time efficiencies enabled by survey design features that eliminated the need for participants to repeat redundant job information. This time efficiency was realized because of moderately high concordance between the current and longest-held jobs – a result consistent with findings from the 2010 National Health Interview Study [33]. The survey design strategy was time-efficient, as confirmed by the survey administration time estimates, but it introduced complexity prior to data collection by the need to program (and test) elaborate skip patterns into the CATI system.

The survey design strategy also introduced complexity after data collection when it became necessary to assign current or longest-held jobs to the enrollment period (see Additional file 1). Because administration of the occupational survey lagged participant enrollment by a median of 6.5 years, participants retrospectively reported enrollment job information. To account for the possibility of recall error and to minimize it, all retrospectively reported job data were verified against logic criteria (e.g., date matching) to minimize the chance for error in the enrollment job assignment. Because employment status had not been obtained at enrollment for the full cohort due to a lag in the inclusion of this variable into the enrollment interview, 33% of enrollment job assignments were based solely on the retrospective report. Enrollment job information was set to missing for 11% of participants who reported that they were employed at enrollment, but job information provided did not date-match their enrollment date.

Because life course exposure to adverse working conditions can be an important determinant of chronic health later in life [34], it is best to collect participants’ current and historical occupational data at the time of enrollment into health studies, and to then routinely update this information during the follow-up period. The occupational history should be as complete as study resources permit, with the minimum suggested data for each job to include industry type, job title (wage employed)/kind of work (self-employed), main job activities or duties, and supervisory responsibilities (wage employed)/number of employees (self-employed). For each reported job we found that it took 94 to 139 seconds (1.6 to 2.3 minutes) to collect this information by CATI.

## Conclusions

The feasibility of conducting high-quality occupational data collection and I/O coding for a large heterogeneous population-based sample was demonstrated. We found that training of interview staff was important to ensuring that narrative responses for industry and occupation were adequately specified for subsequent coding. Relevant training materials have been developed by NIOSH, which are available for download from the NIOSH website [26,27]. A computer-assisted coding system developed at NIOSH was used to improve the time-efficiency of I/O coding. Since coding for this study was completed, the knowledge-base of this coding system has been enhanced and is now capable of automatically assigning codes to some records at the time of data import. This enhanced system, known as the NIOSH Industry and Occupation Computerized Coding System (NIOCCS) is now publically accessible through the NIOSH website [28], enabling future studies to benefit from even greater time efficiencies than was reported here for the REGARDS Study. Because the U.S. Census index of industries and occupations, and associating coding systems such as NIOCCS, are only available in English at this time, researchers planning studies with non-English speaking participants need to plan for the additional time and expense of translation prior to coding. Occupational data now augment the extensive clinical data collected during initial enrollment and follow-up for the REGARDS cohort, enabling future analyses on the association between under-studied modifiable workplace risk factors and chronic health conditions such as stroke, heart disease, and cognitive decline.

## Abbreviations

CATI: Computer-Assisted Telephone Interview; CDC: Centers for Disease Control and Prevention; CHD: Coronary heart disease; CVD: Cardiovascular disease; I/O: Industry and Occupation; NIH: National Institutes of Health; NINDS: National Institute for Neurological Disorders and Stroke; NIOCCS: NIOSH Industry and Occupation Computerized Coding System; NIOSH: National Institute for Occupational Safety and Health; REGARDS: REasons for geographic and racial differences in stroke; SES: Socioeconomic status; SRU: Survey Research Unit; UAB: University of Alabama at Birmingham.

## Competing interests

The authors declare that they have no competing interests.

## Authors’ contributions

LAM and VJH conceived the occupational ancillary study and prepared all IRB documents. LAM, LP and VJH developed the survey and data collection protocols. MJH performed data management and all data analyses. All authors contributed to writing and revising this manuscript. All authors read and approved the final manuscript.

## Pre-publication history

The pre-publication history for this paper can be accessed here:

http://www.biomedcentral.com/1471-2458/14/142/prepub

## Supplementary Material

Additional file 1 Enrollment Job Ascertainment for the Occupational Ancillary Study Sample. Click here for file
