# Supplementary material for: Methods and feasibility of collecting occupational data for a large population-based cohort study in the United States: the reasons for geographic and racial differences in stroke study
Source: BMC Public Health. 2014 Feb 10;14:142. doi: 10.1186/1471-2458-14-142 (PMC3933294; doi:10.1186/1471-2458-14-142)
Supplement: Additional file 1 — Enrollment Job Ascertainment for the Occupational Ancillary Study Sample. [file 1471-2458-14-142-S1.pdf]

Additional file 1: Enrollment Job Ascertainment for the Occupational Ancillary Study Sample

Background

A principal aim of the occupational ancillary study was to collect occupational data among REGARDS cohort participants to enable researchers to examine associations between occupational exposures (e.g., job strain, shift work) and health outcomes, adjusted for individual risk factors and confounders. As part of the ancillary study, participants were asked to provide details of their current job (if currently employed), the job that they held the longest in their lifetime (if ever employed outside the home), and the job held at the time of enrollment in the REGARDS study (if employed at that time). To shorten administration time, and to avoid having participants repeat job details unnecessarily, participants were asked to provide details about their longest-held job only if this job was different from their current job and details about the job they held at enrollment only if this job was different from their current and longest-held jobs. Thus, each participant reported characteristics of up to three jobs – current, longest held, and enrollment. For analyses including all jobs held at enrollment, it was necessary to select among these three job types to ascertain the job held at enrollment for each participant. Because administration of the occupational survey lagged participant enrollment (and the baseline clinical exam) by a median of 6.5 years (range 3.5 to 9.5 years), participants retrospectively reported information (employment status and job characteristics) about their enrollment job. Correspondingly, all retrospectively reported job data were verified against prescribed criteria to ensure that decisions made in the

ascertainment or assignment of the enrollment job accounted for potential sources of response error and otherwise satisfied logic criteria (e.g., date matching).

This report documents data response patterns (or “scenarios”) related to enrollment employment status; data verification procedures; consideration of sources of response error; and decision rules applied in enrollment job ascertainment. These procedures are illustrated graphically in Figure A1. The first step in the process of ascertainment of the enrollment job involved examining agreement between two reports of enrollment employment status. Table A1 shows the cross-tabulation of enrollment employment status initially reported at the enrollment interview and subsequently reported at the time of the occupational survey for all participants completing the occupational module through March 25, 2013. Since the employment status question was added to the enrollment interview in late July 2004, about 18 months after the start of enrollment, this information is missing for 38% of the cohort. Among participants with both sources of enrollment employment status (n=11,554), overall concordance was 87% (10083/11554). The second step involved performing logic checks to verify whether employment dates for the job reportedly held at enrollment (i.e., current, longest-held, or enrollment) actually coincided with the enrollment date. For example, when participants indicated that their enrollment job was the same as their current job we expected the current job to have started no later than the enrollment year (i.e., current job start year ≤ enrollment year).[1] Likewise, when participants indicated that their enrollment job was the same as their longest-held job, we expected the longest-held job to have started

[1] A participant who enrolled in the REGARDS study in 2006 and reported at the occupational survey in 2012 details of their current job that began *in 2005* would pass this logic check; whereas, a participant who enrolled in 2008 and reported at the occupational survey in 2012 details of their current job that began *in 2009* would fail this logic check.

before the enrollment interview and to have stopped after the enrollment interview (i.e., longest-held job stop year – longest-held job tenure ≤ enrollment year ≤ longest-held job stop year).[2] Similar methods were used to verify that employment dates for the reported enrollment job coincided with the enrollment date.

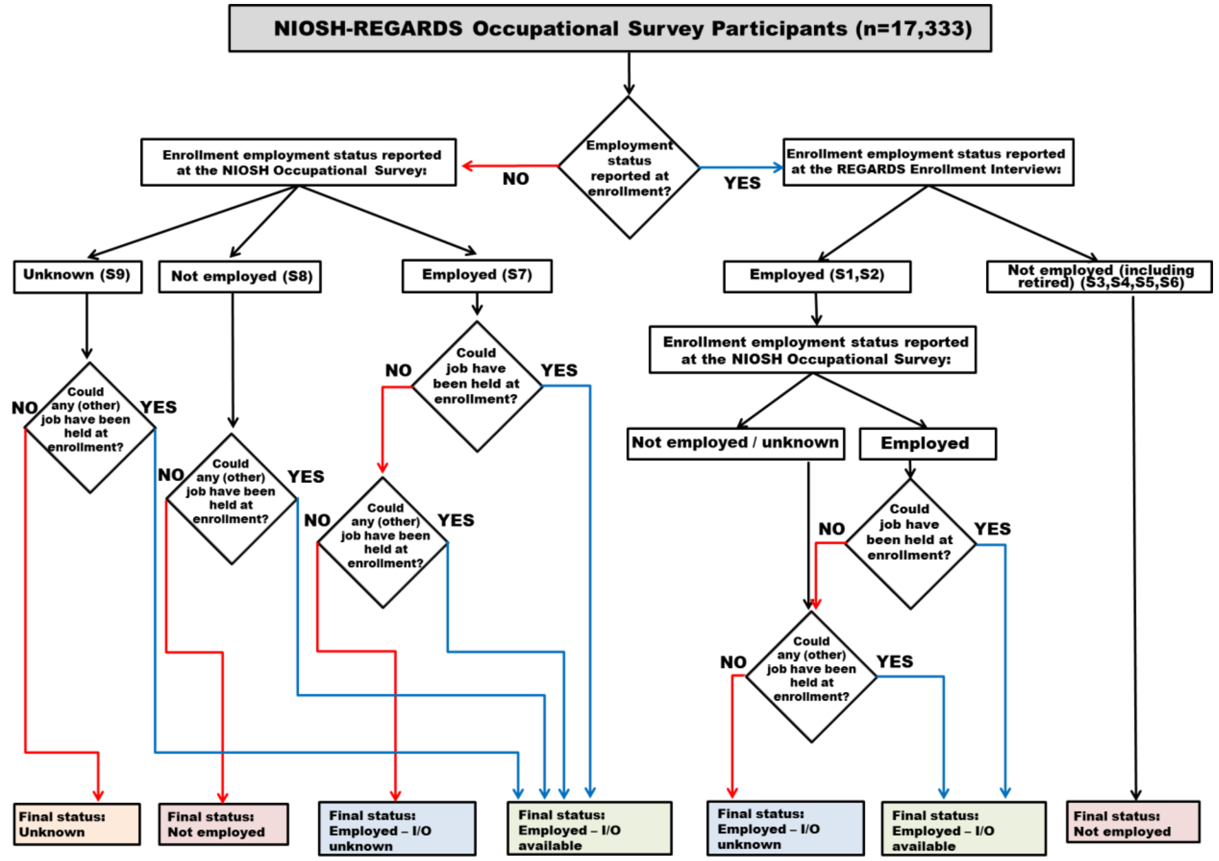

Figure A1. Illustration of decision rules used to assign the enrollment job. Numbers in parentheses refer to scenario numbers (S#) defined in Table A1.

[2] A participant who enrolled in the REGARDS study in 2006 and reported at the occupational survey in 2012 details of their longest-held job that was held for 12 years and ended in 2008 would pass this logic check (i.e., the longest-eld job began in approximately 2008-12=1996 *before* the enrollment year of 2006 and the longest-held job ended in 2008 *after* the enrollment year); whereas, a participant who enrolled in the REGARDS study in 2006 and reported at the occupational survey in 2012 details of their longest-held job that was held for 12 years and ended in 2004 would fail this logic check (i.e., although the longest-held job began in approximately 2004-12=1998 prior to the enrollment year of 2006 it also ended prior to enrollment so it could not have been held at enrollment).

Table A1. Cross-tabulation of enrollment employment status reported first at the time of enrollment and then retrospectively reported at the time of the occupational survey (n=17,333).

Enrollment employment status reported at the enrollment interview [a]	Enrollment employment status retrospectively reported at the occupational survey [b]		
	Working for pay	Not working for pay	Unknown [c]
Employed	4260 (scenario 1)	461 (scenario 2)	25 (scenario 2)
Not retired and not employed [d]	294 (scenario 4)	1365 (scenario 3)	17 (scenario 4)
Retired	716 (scenario 6)	4458 (scenario 5)	35 (scenario 6)
Refused or not available [e]	2632 (scenario 7)	3032 (scenario 8)	38 (scenario 9)

[a] Enrollment interview question: “Are you currently employed for wages, self-employed, out of work (>1 year), out of work (<1 year), homemaker, student, retired, or unable to work?”
[b] Occupational survey question: “At the time you were enrolled in the REGARDS study in [month/year], were you working for pay?” included responses of yes, no, don’t know, and refused.
[c] Don’t know, refused, or otherwise missing data.
[d] Unemployed (out of work, homemaker, student, or unable to work).
[e] Employment status was not part of the original enrollment questionnaire; it was added in late July 2004, after 38% of the cohort had been enrolled.

Suspected sources of error

Recall bias

Because of the time-lag between enrollment and administration of the occupational survey, recall bias may have introduced error in the retrospective reporting of enrollment employment status for some participants. When the retrospective report was not in agreement, we used the employment status reported at the enrollment interview, when it was available. Additionally, since recall bias could have introduced errors in participant reports of job dates/tenure, we relaxed the date requirements for a job to have coincided with the enrollment interview by

(a) allowing the current job to have started before or within 2 years of the enrollment interview (i.e., current job start year ≤ enrollment year + 2) and

(b) allowing the longest-held (or enrollment) job to have started before or within 2 years of the enrollment interview (i.e., longest-held job stop year – longest-held job tenure ≤ enrollment year + 2) and to have stopped after or within 2 years of the enrollment interview (i.e., enrollment year – 2 ≤ longest-held job stop year).

We selected two years because exact job dates were not provided [i.e., participants reported the start year for current jobs, the stop year (and tenure in years) for the longest held job, and the stop year (and tenure in years) for the job held at enrollment].

Response ambiguity

A second potential source of reporting error involved the possibility that some participants may have been undergoing a phased retirement at the time of enrollment into the study, creating response ambiguity for enrollment employment status. For those who had retired from their usual line of work but were continuing to work for pay, responses of “employed for wages” or “self-employed” were expected, but a response of “retired” is also plausible. To permit us to examine the size of this potential source of error, we retained the “retired” category when examining enrollment employment status response patterns (Table A1), rather than grouping retired individuals with those who were not employed.

Response scenarios

To facilitate further examination of employment status reporting discrepancies and potential sources of response bias, the 12 cells in Table A1 were classified into 9 unique participant response scenarios. Each response scenario is described below, along with decision rules applied in the determination of employment status at enrollment as well as the enrollment job assignment.

Scenario 1: Participant's response patterns agree; enrollment employment status was employed (n=4260)

When participants initially reported at the enrollment interview that they were employed, and later (at the time of the occupational survey) also reported that they had been employed at enrollment (n=4260), we were confident that the participant was indeed employed at enrollment. However, we still needed to verify whether the specific job the participant indicated was held at enrollment (i.e., current, longest-held, or enrollment) had employment dates that coincided with the enrollment interview date. For some participants (n=103, 2.4%), missing job dates/tenures precluded verification; for these, job characteristics of the indicated job were assumed to be correct. Among the remaining participants, the "relaxed" date verification procedure (described above) confirmed the enrollment job assignment for many participants (n=3836, 90%). For some participants (n=97, 2.3%), the enrollment interview date corresponded to a different job than the one they reported as being held at enrollment (e.g., a subject may have reported that their current job was held at enrollment, but inspection of the job dates indicated that the current job actually started sometime after enrollment and the

longest-held job coincided with the enrollment interview date); for these participants, enrollment job characteristics were assigned using the job that coincided with the enrollment interview date. When no reported job coincided with the enrollment interview date (n=224, 5.3%), the enrollment employment status was retained as “employed,” but enrollment job characteristics were set to missing/unknown. Table A2 summarizes the decision basis for assigning enrollment employment status and job characteristics.

Table A2. Decision basis for assigning enrollment employment status among the occupational ancillary study sample (n=17,333).

Decision basis for assigning enrollment employment status	Assigned enrollment employment status			
	Working for pay		Not working for pay	Unknown
	Job characteristics available	Job characteristics not available		
Concordant reports of “employed”				
Use the indicated job that matched the enrollment interview date for the enrollment job	**3836$_{S1}$** [a,b]			
Use the indicated job that is *assumed* to match the enrollment interview date for the enrollment job (match cannot be verified due to missing job dates/tenure)	*103*$_{S1}$ [c]			
Use a *different* reported job that matched the enrollment interview date for the enrollment job	*97*$_{S1}$			
No job match to enrollment interview date		224$_{S1}$ [d]		
Relied on concurrent report of “employed”, ignored retrospective report of “not employed”				
Use a *different* reported job that matched the enrollment interview date for the enrollment	*171*$_{S2}$			

job				
No job match to enrollment interview date		315_{S2}		
Concordant reports of “not employed”				
Never employed outside the home			**$159_{S3}+65_{S5}$**	
All reported jobs consistent with not employed status at enrollment interview			**$1014_{S3}+3940_{S5}$**	
All reported jobs *assumed* to be consistent with not employed status at enrollment interview (this cannot be verified due to missing job dates/tenure)			*$144_{S3}+306_{S5}$*	
Ignore other reported job(s) that matched to enrollment interview date			$48_{S3}+147_{S5}$	
Relied on concurrent report of “not employed”, ignored retrospective report				
All reported jobs consistent with not employed status at enrollment			**$70_{S4}+166_{S6}$**	
All reported jobs *assumed* to be consistent with not employed status at enrollment (this cannot be verified due to missing job dates/tenure)			*$40_{S4}+83_{S6}$*	
Ignore indicated job that matched enrollment interview date			486_{S6}	
Ignore other reported job(s) that matched enrollment interview date			$201_{S4}+16_{S6}$	
Relied on retrospective report of “employed”, concurrent report not available				
Use the indicated job that matched the enrollment interview date for the enrollment job	**2175_{S7}**			
Use the indicated job that is *assumed* to match the enrollment interview date for the enrollment job (match cannot be	*109_{S7}*			

verified due to missing job dates/tenure)				
Use a *different* reported job that matched the enrollment interview date for the enrollment job	*64$_{S7}$*			
No job match to enrollment interview date		284$_{S7}$		
Relied on retrospective report of “not employed”, concurrent report not available				
Never employed outside the home			**51$_{S8}$**	
All reported jobs *assumed* to be consistent with not employed status at enrollment (this cannot be verified due to missing job dates/tenure)			*233$_{S8}$*	
All reported jobs consistent with not employed status at enrollment			**2600$_{S8}$**	
Ignore retrospective report and used a different reported job that matched the enrollment interview date for the enrollment job	*148$_{S8}$*			
No information about enrollment employment status				
Use a *different* reported job that matched the enrollment interview date for the enrollment job	*6$_{S9}$*			
No job match to enrollment interview date				32$_{S9}$
**Total**	6709 (39%)	823 (5%)	9769 (56%)	32 (<1%)

[a] Subscript refers to the scenarios defined in Table A1.
[b] **Bold text** indicates that the reported job information was consistent with the reported enrollment status and able to be verified.
[c] *Italic text* indicates that the reported job information was in some way inconsistent, or could not be verified due to missing job dates/tenure.
[d] Plain text indicates that the job information was not provided or that provided job information was ignored.

Scenario 2: Participant's retrospective response does not confirm employed status reported at enrollment (n=486)

When participants reported at the enrollment interview that they were employed, but later reported that they had not been employed at enrollment (n=461) or that their enrollment employment status was unknown (n=25), we assumed that the participant really was employed at enrollment because they had a concurrent report. We then performed "relaxed" date verifications to determine if dates for any reported job coincided with the enrollment interview. For some participants (n=171, 35%), another reported job had employment dates that coincided with the enrollment interview date and this date-matched job was assigned as the enrollment job. The remaining participants were assumed to have been working at enrollment, but their enrollment job characteristics were set to missing/unknown because no reported job had dates that coincided with the date of the enrollment interview (n=315, 65%).

Scenario 3: Participant's response patterns agree; enrollment employment status was not employed (n=1,365)

When participants reported at the enrollment interview that they were not employed (but not retired) and later reported that they had not been employed at enrollment (n=1365), we were confident that the participant was indeed not employed at enrollment. Some of these participants reported never having been employed for wages since the age of 25 years (n=159, 12%). For other participants, as a check, we used the strict date verification procedure to verify that no other reported job could have been held at enrollment. In most cases, the enrollment interview date did not coincide with any

reported job (n=1014, 74%); but this could not be verified for some participants (n=144, 11%) due to missing job dates/tenure. Some participants, however, provided job dates/tenure for a job that may have been held at enrollment (n=48, 4%); for these, we retained the unemployed enrollment employment status and attributed the date matching to recall bias (error) in the reported job dates/tenure for the other job.

Scenario 4: Participant's retrospective response does not confirm unemployed status reported at enrollment (n=311)

When participants reported at the enrollment interview that that they were not employed (but not retired) and later reported that they had been employed at enrollment (n=294) or this was unknown (n=17), we assumed that the participant really was not employed at enrollment and ignored any enrollment job information reported. For a majority of these participants (n=201, 65%), the enrollment interview date coincided with the reported job dates/tenure using the strict date verification procedure, but these findings were attributed to error (recall bias). For the remainder, the enrollment interview date did not coincide with the reported job dates/tenure (n=70, 22%) or missing job dates/tenure precluded verification (n=40, 13%).

Scenario 5: Participant's retrospective response is consistent with retirement status reported at enrollment (n=4,458)

When participants reported at the enrollment interview that that they were retired and later reported that they had not been employed at enrollment (n=4458), we retained the enrollment employment status as not employed (retired). Some of these participants

also reported never having been employed for wages since the age of 25 years (n=65, 1%). For other participants, as a check, we attempted to verify that no reported jobs could have been held at enrollment using the (strict) date verification procedure. In most cases, no reported job dates coincided with the enrollment interview date (n=3940, 88%); but this could not be verified for some participants (n=306, 8%) due to missing job dates/tenure. Some participants, however, provided job dates/tenure for a job that coincided with the enrollment interview date (n=147, 3%); for these, we retained the unemployed (retired) enrollment employment status and attributed the date verification finding to recall bias in the job dates/tenure for the other reported jobs.

Scenario 6: Participant's retrospective response does not confirm retired status reported at enrollment, possibly due to phased retirement (n=751)

When participants reported at the enrollment interview that they were retired and later reported that they had been employed at enrollment (n=716) or that their enrollment employment status was unknown (n=35), we assumed that the participant really was retired (and not employed) at enrollment and ignored the retrospective report. To evaluate the extent that these participants may have been semi-retired (i.e., working for pay in a post-retirement job), we used the relaxed date verification procedures to determine if any reported job could have been held at enrollment. For some participants (n=83, 11%), missing job dates/tenures precluded verification; for other participants (n=166, 22%), no jobs coincided with the enrollment interview date. However, for many participants, either the indicated job (n=486, 65%) or another reported job (n=16, 2%) was consistent with its being held at enrollment. We elected to not assign these date-

matched jobs to enrollment in favor of maintaining the concurrent report of retired at enrollment.

Scenario 7: Participant's retrospective response of employed at enrollment cannot be verified due to missing data at enrollment (n=2,632)

For participants that were not asked about their employment status during the enrollment interview, but later reported that they were employed at enrollment (n=2632), we relied on the retrospective report and assigned an enrollment employment status of "employed". For some participants (n=109, 4%), missing job dates/tenures precluded verification; for these, job characteristics of the indicated job were assumed to be correct. The "relaxed" date verification procedure confirmed the enrollment job assignment for many participants (n=2175, 83%). For some participants (n=64, 2%), the enrollment interview date corresponded to a different job than the one the participant reported as being held at enrollment; for these participants, enrollment job characteristics were assigned using the job that coincided with the enrollment interview date. When no reported job coincided with the enrollment interview date (n=284, 11%), the enrollment employment status was retained as "employed," but enrollment job characteristics were set to missing/unknown.

Scenario 8: Participant's retrospective response of not employed at enrollment cannot be verified due to missing data at enrollment (n=3,032)

For participants not asked about their employment status during the enrollment interview but later responded that they were not employed at enrollment (n=3032), we

initially relied on the retrospective report and assigned an enrollment employment status of “not employed”. Indeed, for many of these participants (n=2600, 85%) we verified that the enrollment interview date did not coincide with another reported job using the strict date verification procedure; other participants reported never being employed for wages (n=51, 2%) or missing job dates precluded verification (n=233, 8%). However, when the enrollment interview date did coincide with another reported job (n=148, 5%), we assigned an enrollment employment status of “employed”. In this latter situation, we attributed the error to recall bias in the report of enrollment employment status and used the job characteristics of the date-matched job.

Scenario 9: Participant’s response patterns are incomplete due to missing data from both sources (n=38)

Finally, both reports of enrollment employment status were unknown for a small number of participants (n=38). We assigned an enrollment employment status of “unknown” to all of these with the exception of a few participants (n=6) for which the enrollment interview date coincided with a reported job using the relaxed date verification procedures. In this situation, we assigned an enrollment employment status of “employed” and used the job characteristics of the date-matched job.

Summary

Enrollment employment status was assigned as employed for a total of 7532 participants (43%); of these, enrollment job characteristics will be unknown for 823 participants. More than half of the participants (n=9769) will be treated as not employed

at enrollment. A few participants (n=32) will have unknown enrollment employment status.

Enrollment employment status data was consistent and able to be verified for a majority of participants (n=14076, 81%). Information was in some way inconsistent (e.g., concurrent and retrospective reports were discordant) or not able to be verified (e.g., due to missing job dates/tenure) for 1504 participants (9%). Information was not provided (e.g., when concurrent report was employed, but retrospective report was not employed) or ignored (e.g., when concurrent report was not employed, but retrospective report was employed) for 1753 participants (10%).
